# COVID-19 in the Perioperative Period of Cardiovascular Surgery: the Brazilian Experience

**DOI:** 10.21470/1678-9741-2021-0960

**Published:** 2021

**Authors:** Walter J. Gomes, Isadora Rocco, Wallace S. Pimentel, Aislan H. B. Pinheiro, Paulo M. S. Souza, Luiz A. A. Costa, Marjory M. P. Teixeira, Leonardo P. Ohashi, Caroline Bublitz, Isis Begot, Rita Simone L Moreira, Nelson A. Hossne Jr, Guilherme F. Vargas, João Nelson R. Branco, Carlos A. Teles, Eduardo A. S. Medeiros, Camila Sáfadi, Amândio Rampinelli, Leopoldo Moratelli Neto, Anderson Rosa Rosado, Franciele Kuhn Mesacasa, Ismael Escobar Capriata, Rodrigo Coelho Segalote, Deborah Louize da Rocha Vianna Palmieri, Amanda Cristina Mendes Jardim, Diego Sarty Vianna, Joaquim Henrique de Souza Aguiar Coutinho, João Carlos Jazbik, Henrique Madureira da Rocha Coutinho, Gustavo Kikuta, Zely Sant'Anna Marotti de Almeida, Gibran Roder Feguri, Paulo Ruiz Lucio de Lima, Anna Carolina Franco, Danilo de Cerqueira Borges, Felipe Ramos Honorato De La Cruz, Ulisses Alexandre Croti, Bruna Cury Borim, Carlos Henrique De Marchi, Lilian Goraieb, Karolyne Barroca Sanches Postigo, Fabiano Gonçalves Jucá, Fátima Rosane de Almeida Oliveira, Rafael Bezerra de Souza, Alexandre Cabral Zilli, Raul Gaston Sanchez Mas, Luiz Carlos Bettiati Junior, Ricardo Tranchesi, Ayrton Bertini Jr, Leandro Vieira Franco, Priscila Fernandes, Fabiana Oliveira, Roberto Moraes Jr, Thiago Cavalcanti Vila Nova de Araújo, Otávio Penna Braga, Antônio Cavalcanti Pedrosa Sobrinho, Roberta Tavares Barreto Teixeira, Irla Lavor Lucena Camboim, Eduardo Nascimento Gomes, Pedro Horigushi Reis, Luara Piovan Garcia, Nelson Henrique Goes Scorsioni, Roberto Lago, Solange Guizilini

**Affiliations:** 1 Cardiology and Cardiovascular Surgery Disciplines, Hospital São Paulo, Escola Paulista de Medicina, Universidade Federal de São Paulo, São Paulo, São Paulo, Brazil.; 2 Cardiology Postgraduate Program, Universidade Federal de São Paulo, São Paulo, São Paulo, Brazil.; 3 Escola Paulista de Enfermagem, Universidade Federal de São Paulo, São Paulo, Brazil.; 4 Sociedade Brasileira de Cirurgia Cardiovascular, São Paulo, São Paulo, Brazil.; 5 Instituto de Cardiologia de Santa Catarina, Florianópolis, Santa Catarina, Brazil.; 6 Instituto Nacional de Cardiologia, Rio de Janeiro, Rio de Janeiro, Brazil.; 7 Hospital Universitário Pedro Ernesto, Rio de Janeiro, Rio de Janeiro, Brazil.; 8 Hospital Geral Filantrópico Universitário de Cuiabá, Cuiabá, Mato Grosso, Brazil.; 9 Pediatric Cardiology and Cardiovascular Surgery Service at Hospital da Criança e Maternidade de São José do Rio Preto, São José do Rio Preto, São Paulo, Brazil.; 10 Hospital de Messejana Dr. Carlos Alberto Studart Gomes, Fortaleza, Ceará, Brazil.; 11 Hospital de Caridade São Vicente de Paulo, Jundiaí, São Paulo, Brazil.; 12 Hospital Geral de Pirajussara, Taboão da Serra, São Paulo, Brazil.; 13 Hospital Universitário Nova Esperança, João Pessoa, Paraíba, Brazil.; 14 Hospital Regional de Sorocaba Dr. Adib Domingos Jatene, Sorocaba, São Paulo, Brazil.

**Keywords:** COVID-19, SARS-CoV-2, Cardiac Surgical Procedures, Thoracic Surgery, Comorbidity

## Abstract

**Introduction:**

We investigated the clinical course and outcomes of patients submitted to cardiovascular surgery in Brazil and who had developed symptoms/signs of coronavirus disease 2019 (COVID-19) in the perioperative period.

**Methods:**

A retrospective multicenter study including 104 patients who were allocated in three groups according to time of positive real time reverse transcriptase-polymerase chain reaction (RT-PCR) for the SARS-CoV-2 (severe acute respiratory syndrome coronavirus 2): group 1, patients who underwent cardiac surgery > 10 days after positive RT-PCR; group 2, patients with a positive RT-PCR within 10 days before or after surgery; group 3, patients who presented positive RT-PCR > 10 days after surgery. The primary outcome was mortality and secondary outcomes were postoperative complications, intensive care unit (ICU) length of stay, and postoperative days of hospitalization.

**Results:**

The three groups were similar with respect to age, the European System of Cardiac Operative Risk Evaluation score, and comorbidities, except hypertension. Postoperative complications and death were significantly higher in groups 2 and 3 than in group 1, and no significant difference between groups 2 and 3 was seen. Group 2 showed a high prevalence of surgery performed as an urgent procedure. Although no significant differences were observed in ICU length of stay, total postoperative hospitalization time was significantly higher in group 3 than in groups 1 and 2.

**Conclusion:**

COVID-19 affecting the postoperative period of patients who underwent cardiovascular surgery is associated with a higher rate of morbidity and mortality. Delaying procedures in RT-PCR-positive patients may help reduce risks of perioperative complications and death.

**Table:** 

Abbreviations, acronyms & symbols				
AIC	= Akaike’s information criterion		HBP	= High blood pressure
AKI	= Acute kidney injury		ICU	= Intensive care unit
ARDS	= Acute respiratory distress syndrome		IQR	= Interquartile range
BMI	= Body mass index		MI	= Myocardial infarction
CAD	= Coronary artery disease		OR	= Odds ratio
CI	= Confidence interval		RNA	= Ribonucleic acid
CKD	= Chronic kidney disease		RRT	= Renal replacement therapy
COPD	= Chronic obstructive pulmonary disease		RT-PCR	= Reverse transcriptase-polymerase chain reaction
COVID-19	= Coronavirus disease 2019		SARS-CoV-2	= Severe acute respiratory syndrome coronavirus
EuroSCORE	= European System for Cardiac Operative Risk Evaluation		SD	= Standard deviation
GzLM	= Generalized linear models			

## INTRODUCTION

The coronavirus disease 2019 (COVID-19) pandemic has spread and stricken the entire world in an unimaginable proportion, with the number of confirmed cases rapidly increasing and leaving in its wake millions of deaths. Besides triggering a devastating socioeconomic crisis, the COVID-19 pandemic is inflicting a global health disaster, damaging the capability to manage affected people, and disrupting established care paths and the extent to provide the standard of care for critically ill patients. The COVID-19 pandemic has changed the practice of surgery worldwide; to safeguard resources, treatments are deferred or alternative strategies have been advised, leading to a dire reduction of surgical volumes^[1]^.

The toll taken by COVID-19 on patients who underwent surgery at this time is drastic. An international multicenter study assessing 1,128 patients who had surgery and postoperatively developed COVID-19 revealed a 23.8% 30-day mortality, and pulmonary complications occurred in 51.2%; the 30-day mortality in these patients was 38.0%, accounting for 82.6% of all deaths^[2]^. Higher rates of morbidity and mortality in patients with COVID-19 are related to underlying conditions such as hypertension, coronary artery disease (CAD), diabetes, and chronic renal disease - risk factors commonly associated with patients requiring cardiovascular surgery^[3,4]^.

The potential impact of concomitant COVID-19 on patients undergoing cardiovascular procedures remains poorly characterized, and further data are relevant and crucial for determining critical patient-centered surgical decision making.

This multicenter study aimed to investigate the clinical course and outcomes of patients submitted to cardiovascular surgery in Brazil and who had developed symptoms/signs of COVID-19 in the perioperative period of cardiovascular surgery.

## METHODS

This retrospective multicenter cohort study collected data from adult subjects (≥ 18 years old) who underwent cardiovascular surgery and had confirmed COVID-19 in the perioperative period, between March 10, 2020, and July 16, 2021, at 11 referral centers across Brazil.

In the beginning, an invitation letter was sent to cardiac surgery centers in Brazil to participate in this study voluntarily through the Brazilian Society of Cardiovascular Surgery. Those agreeing to share their information signed a specific informed consent form, involving institutions distributed across the Brazilian territory. This study was approved by Institutional Ethics Committees (# 4.236.309) and a signed consent form was obtained from each subject. The study followed the Strengthening the Reporting of Observational Studies in Epidemiology (or STROBE) guidelines for reporting observational studies^[5]^.

Data were retrospectively collected following a tailored protocol including key information for patient demographics, risk factors, operative variables, and postoperative clinical outcomes.

The 11 participating centers are well distributed among the following regions of the country: Southeast (n=7), Northeast (n=2), South (n=1), and Central-West (n=1).

Patients were screened according to clinical history and development of symptoms/signs compatible with COVID-19, tested positive for COVID-19, and were diagnosed according to the World Health Organization Interim Guidance Document^[6]^. Laboratory confirmation of severe acute respiratory syndrome coronavirus 2 (SARS-CoV-2) infection was carried out by quantitative reverse transcriptase-polymerase chain reaction (RT-PCR) on samples from the respiratory tract.

In this analysis, patients were allocated in three groups according to the period of positive RT-PCR test in relation to the surgery time: group 1, patients who underwent cardiac surgery > 10 days after the positive RT-PCR test; group 2, patients with a positive RT-PCR test within 10 days before or after surgery; group 3, patients who presented with positive RT-PCR test > 10 days after surgery. This timeframe was selected based on current evidence that persons with mild to moderate COVID-19 may shed replication-competent SARS-CoV-2 for up to 10 days following symptom onset^[7]^.

The definitions of procedural classifications (emergent, urgent, elective, and non-urgent procedures) followed the recommendations of the COVID-19 Guidelines for Triage of Cardiac Surgery Patients, issued by the American College of Surgeons^[8]^. Whenever missing or uncertain records were recognized, direct communication with patients and their families helped solve the matter. Patients with inconsistency of clinical history and/or confirmation of RT-PCR for SARS-CoV-2 were excluded from the analysis.

Early into the pandemic, the diagnosis protocol for COVID-19 was not standardized across the country and the centers and swab tests were collected anytime between one to seven days before surgery.

### Clinical Data and Outcomes

Primary endpoint was mortality rate and secondary outcomes were postoperative complications, intensive care unit (ICU) length of stay, and days of postoperative hospitalization. The European System for Cardiac Operative Risk Evaluation (EuroSCORE) II of all included subjects was calculated.

The assessed postoperative complications were acute kidney injury (*i.e*., serum creatinine ≥ 2.0 mg/d and anuria for 12 hours or urine output < 0.3 mL/kg/h for six consecutive hours, according to the KDIGO clinical practice guidelines^[9]^, need for renal replacement therapy, acute respiratory distress syndrome (ARDS) according to the Berlin definition^[10]^, cardiogenic shock, and pneumonia. The presence of at least one of the postoperative complications was measured as a secondary outcome.

### Statistical Analysis

Categorical data were presented as absolute and relative frequency (n and %, respectively) and initially compared with Fisher's exact test. Continuous and discrete variables were presented as mean ± standard deviation and median with interquartile range, respectively. Generalized linear models (GzLM) were performed to compare groups among quantitative dependent variables according to their distribution aiming at minor residuals and best Akaike's information criterion. Logistic regressions were performed separately for qualitative outcomes (postoperative complications and mortality) to investigate potential odds ratio (OR) among groups. GzLM and logistic regressions were adjusted by the type of surgery, *i.e*., elective, urgency, or emergency. Graphs and all analyses were performed using the statistical software Jamovi, version 1.6.23.0 plus Rj Editor.

## RESULTS

**Fig. 1:**
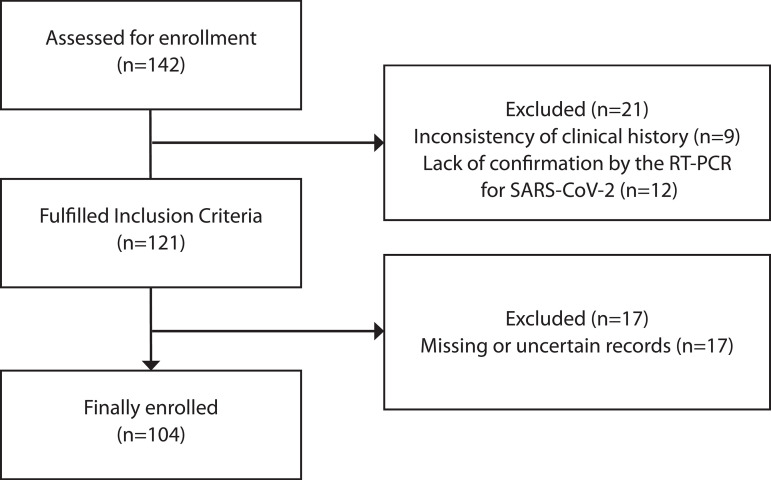
Flowchart of patients in the study. RT-PCR=reverse transcriptase-polymerase chain reaction; SARS-CoV-2=severe acute respiratory syndrome coronavirus 2.

One hundred and four patients met the inclusion criteria. The flowchart of patients' baseline characteristics enrolled in the study is illustrated in Figure 1. Demographic and clinical characteristics of patients are presented in Table 1.

**Table 1:** Patients' demographic and clinical characteristics.

	TOTAL (N=104)					
	%	Mean	±	SD	Median	IQR
Age (years)		60.7	±	12.8	62	(54.5-70.0)
Sex (male)	63.7					
Height (cm)		166	±	9.10	166	(160-170)
Weight (kg)		76.0	±	18.2	74.5	(62.2-84.3)
BMI (kg/m^2^)		27.5	±	5.48	26.5	(23.5-31.2)
Blood type						
A	42.9					
B	1.4					
AB	17.1					
O	38.6					
Rh factor						
Positive	12.7					
Negative	87.3					
HBP	75.0					
Diabetes	40.0					
Active smoking	44.8					
CAD	66.3					
Stroke	5.2					
CKD	12.2					
COPD	11.2					
Previous MI	18.0					
EuroSCORE II		3.91	±	3.53	2.46	(1.28-5.35)
Surgery timing						
Elective	70.1					
Urgency	26.2					
Emergency	3.7					
Operation time (min)		239	±	87.7	238	(201-295)
Pump time (min)	87.5	82.3	±	46.1	83	(56-100)
Mean aortic clamping time (min)	87.5	67.7	±	38.4	68	(47-85.3)
Δ COVID-19/surgery time		-15.2	±	47.3	0	(-33.5-9.0)
COVID-19 symptoms/signs						
Dry cough	23.2					
Fever	25.5					
Fatigue	29.8					
Dyspnea	43.2					
Desaturation	44.2					
Headache	4.2					
Diarrhea	3.2					

BMI=body mass index; CAD=coronary artery disease; CKD=chronic kidney disease; COPD=chronic obstructive pulmonary disease; COVID-19=coronavirus disease 2019; EuroSCORE II=European System for Cardiac Operative Risk Evaluation; HBP=high blood pressure (hypertension); IQR=interquartile range; MI=myocardial infarction; SD=standard deviation

The mean age was 60.7±12.8 years, 63.7% were males. Overweight (body mass index [BMI] = 27.5±5.48) patients, and A and O blood types (42.9% and 38.6%, respectively) were more prevalent (Table 1). 70.1% of patients underwent elective cardiac surgery with different types of procedures, mostly on-pump (87.5%).

Table 2 reveals patients' characteristics according to group allocation based on the timeframe between the surgery date and the positive RT-PCR SARS-CoV-2 test. Groups were similar with respect to age, BMI, EuroSCORE II, and comorbidities, except hypertension. Group 2 showed a high prevalence of surgery performed as an urgent procedure (*P*<0.001), which was adjusted for confounding variables in the logistic regression analysis.

**Table 2:** Patients' characteristics according to group allocation.

	Group 1 (N=45)				Group 2 (n=37)				Group 3 (N=22)				
	%	Mean ± SD	Median	IQR	%	Mean ± SD	Median	IQR	%	Mean±SD	Median	IQR	*P*-value
Age (years)		57.5±13.6	59.0	48 - 66		63.2±10.8	64.5	55.3 - 70.0		63.3 ± 13.9	63.5	58 - 74	0.075^b^
Sex (male)	64.4				73.7				50.0				0.193^c^
Height (cm)		167±8.66	166	162 - 170		165±6.88	168	160 - 170		167 ± 12.7	168	161 - 172	0.779^a^
Weight (kg)		76.9±16.2	76.5	65 - 90		73.6±18.4	70.0	60.8 - 80.0		78.4 ± 15.8	75.0	70 -80	0.631^a^
BMI (kg/m^2^)		26.9±5.75	26.4	22 - 30		27.2±4.99	25.1	23.4 - 31.2		29.2 ± 5.2	29.7	26 - 31	0.511^a^
Blood type													0.891^c^
A	43.8				50.0				31.3				
B	3.1				0.0				0.0				
AB	15.6				18.2				18.8				
O	37.5				31.8				50.0				
Rh factor													0.416^c^
Positive	9.4				21.7				6.3				
Negative	90.6				78.3				93.8				
HBP	64.4				86.1				86.4				0.043^c^
Diabetes	37.8				38.9				40.9				0.970^c^
Active smoking	44.2				47.1				54.5				0.733^c^
CAD	59.1				68.4				81.8				0.201^c^
Stroke	7.0				5.7				0				0.717^c^
CKD	9.1				20.0				4.5				0.191^c^
COPD	9.1				14.3				13.6				0.727^c^
Previous MI	60.5				40.5				59.1				0.170^c^
EuroSCORE II		2.89±2.80	1.89	01/mar		4.93 ± 3.85	4.18	2.05 - 7.53		4.09 ± 3.95	1.80	1 - 6	0.078^a^
Surgery timing													<0.001^c^
Elective	86.7				45.9				77.3				
Urgency	13.3				45.9				22.7				
Emergency	0.0				8.1				0.0				
Δ RT-PCR+ to surgery time		-48.4±51.6	-35	-70		4.74 ± 3.36	5.00	02/ago		23.7 ± 17.3	16.0	12 - 28	<0.001^a^

BMI=body mass index; CAD=coronary artery disease; CKD=chronic kidney disease; COPD=chronic obstructive pulmonary disease; EuroSCORE II=European System for Cardiac Operative Risk Evaluation; HBP=high blood pressure; IQR=interquartile range; MI=myocardial infarction; RT-PCR=reverse transcriptase-polymerase chain reaction; SD=standard deviation

a Generalized linear model with gamma distribution

b General linear model with Gaussian distribution

c Fisher's exact test

Postoperative complications were more frequent in groups 2 and 3 than in group 1 - OR=4.45 (95% confidence interval [CI] = 1.64 - 12.87) and OR=3.44 (95% CI = 1.20 - 10.42), respectively (Table 3). Although no significant differences were observed in ICU length of stay, total postoperative hospitalization time was significantly higher in group 3 (Table 3) than in group 1 and group 2 (*P*=0.005 and *P*=0.04 at Bonferroni post hoc test, respectively).

**Table 3:** Clinical outcomes according to group allocation.

Clinical outcomes	Total	Group 1	Group 2	Group 3	*P*-value
	(n=104)	(n=45)	(n=37)	(n=22)	
Vasoactive drugs use (%)	81.9	75.6	86.8	86.4	0.369
ICU length of stay (days)	6 (4 - 11)	5 (4 - 8)	8 (4 -12)	7.5 (5 - 15.5)	0.310^a^
Total postoperative days	11.5 (6 - 24)	7 (5 - 14)	12 (8 - 23)	24 (20 - 41)*	< 0.001^a^
Postoperative complications (%)	52.4	33.3	68.4	63.6	0.003
AKI	25.0	11.4	36.8	31.8	0.016
RRT	9.5	2.2	13.2	18.2	0.044
Cardiogenic shock	23.1	15.9	26.3	31.8	0.294
ARDS	17.1	4.4	18.4	40.9	< 0.001^b^
Pneumonia	42.3	22.7	60.5	50.0	0.002
Deaths	25	2	17	6	< 0.001^b^
Mortality rate	24.0	4.4	45.9	27.3	< 0.001^b^

AKI=acute kidney injury; ARDS=acute respiratory distress syndrome; ICU=intensive care unit; RRT=renal replacement therapy Hospitalization days are presented in median (interquartile range).

a Generalized linear model with gamma distribution with Bonferroni post hoc test

b Fisher's exact test

* P<0.001 compared to group 1

Patients in the groups 2 e 3 presented significantly higher mortality rates than in group 1 (OR=14.85 [95% CI = 3.55 - 102.92], *P*=0.003, and OR=7.77 [95% CI = 1.59 - 57.14], *P*<0.001, respectively, Tables 3 and 4). There were no significant differences between groups 2 and 3 (*P*=0.856). No association was found between ABO blood type and postoperative clinical outcomes.

**Table 4:** Postoperative complications and mortality in regard of group allocation.

Postoperative complications^a^						
**Groups comparison**			**exp(B)**	**95% CI**	**z**	***P*-value**
2	-	1	4.45	1.64 - 12.87	2.86	0.013
3	-	1	3.44	1.20 - 10.42	2.26	0.071
**Mortality^b^**						
**Groups comparison**			**exp(B)**	**95% CI**	**z**	***P*-value**
2	-	1	14.85	3.55 - 102.92	3.65	0.003
3	-	1	7.77	1.59 - 57.14	2.41	0.018

AIC=Akaike's information criterion; CI=confidence interval

Adjusted by the type of surgery, i.e., elective, urgency, or emergency

a AIC=140.90

b AIC=99.19

## DISCUSSION

The key findings of our study reaffirm the inherent risk related to COVID-19 disease occurring throughout the perioperative period of cardiovascular surgery, associated with higher morbidity and mortality. All postoperative complications were significantly more prevalent in groups 2 and 3 than in group 1, except for cardiogenic shock. The group 2 (patients with a positive RT-PCR test within 10 days before or after surgery) had 14 times greater odds of dying than group 1, with a mortality rate of 45.9%, while in group 3, patients who presented with positive RT-PCR test > 10 days after surgery, had three times higher risk of death compared to group 1, with a mortality rate of 27.3%.

Group 2 patients were more likely to undergo surgery as an urgent/emergency procedure, with associated more postoperative complications, which may account for the significantly higher mortality. Group 3 patients required significantly longer hospitalization, had more postoperative complications, and a significantly increased risk of death compared to group 1 (27.3% *vs*. 4.4%, *P*<0.001).

Elderly patients (> 60 years), men, hypertensive, with CAD, with previous myocardial infarction, smokers, and diabetic were highly prevalent in this series, the same risk factors were demonstrated to predispose a worse prognosis in COVID-19^[3,4,11]^.

A steady finding was the relative prevalence of overweight patients in the three groups, reinforcing the relationship between BMI and COVID-19 severity, where impaired antibody production and chronic inflammation favor progression of COVID-19 in overweight subjects. Overweight and obesity were risk factors for invasive mechanical ventilation, hospitalization, and death, particularly among adults aged < 65 years^[12]^.

Although groups 2 and 3 had higher EuroSCORE II than patients in group 1, it failed to reach statistical significance.

The complexity and duration of surgeries (*i.e*., the degree of surgical trauma) play a major role in accelerating and exacerbating the disease progression and severity of latent COVID-19, through an altered immune response. Further to the surgical trauma, cardiopulmonary bypass has been demonstrated to induce a pronounced systemic inflammatory response syndrome, frequently leading to transient immunosuppressive states of different duration and severity^[13-15]^. Such observation applies to our experience, where patients in group 2 developed COVID-19 symptoms within a few days before or after the operation.

The SARS-CoV-2 infection triggers a pro-inflammatory and pro-coagulant state, inducing increased endothelial and microvascular dysfunction, with an elevation of both D-dimer and fibrinogen levels. Increased levels of D-dimer on hospital admission correlate with disease severity or higher risk of mortality^[16]^.

Most deaths from COVID-19 are typically caused by multiple organ dysfunction, related to the triggering of a hyper-inflammation with features of cytokine storm syndrome and associated ARDS. Anti-inflammatory therapy with immunosuppressive steroids inhibiting the hyper-inflammatory immune response in severe COVID-19 pneumonia has improved survival, mostly in those with oxygen requirements^[17]^. Early treatment with heparin suggested a reduction in the risk of death in patients with COVID-19, blunting the pathogenic mechanism associated with systemic hypercoagulability^[18]^.

In our study, the detection of SARS-CoV-2 infection and deferral of surgical intervention proved effective, as seen in group 1 patients, with a delay of 48.4±51.6 days. On the other hand, in group 2 patients, the higher mortality is in agreement with the shorter mean time between the positive RT-PCR test and the date of the operation, with a mean of 4.74±3.36 days, ranging from two to eight days, with more urgent/emergency procedures.

Grounded in our findings, it is clear that the risk of death or severe postoperative complications for COVID-positive patients is lessened as time goes by; a direct relationship of the time interval between the positive RT-PCR and the date of surgery. Given the nationwide impact of COVID on the reduction of elective cardiovascular surgery cases, a growing number of patients were admitted for urgent or emergency intervention and presented an overall more severe risk profile at admission. Not only the operative mortality and morbidity rates were significantly higher but the average hospital resource consumption per patient was accordingly greater, adding for the further reduction of elective cases^[19,20]^.

According to the Centers for Disease Control and Prevention (or CDC), based on current evidence, persons with mild to moderate COVID-19 may shed replication-competent SARS-CoV-2 for up to 10 days following symptom onset. The SARS-CoV-2 ribonucleic acid (RNA) may be detectable in the upper or lower respiratory tract for weeks after illness onset, however, detection of viral RNA does not necessarily mean that the infectious virus is present. Based on existing literature, the incubation period (the time from exposure to development of symptoms) of SARS-CoV-2 ranges from two to 14 days^[7]^.

Dyspnea was the most common early symptom, as it was in reports from China^[3]^, along with arterial oxygen desaturation (SaO_2_ < 93%). Fever was of late presentation, which suggests that this sign may not be a useful criterion for the perioperative suspicion of COVID-19.

A major study has found an association between blood types and the likelihood of respiratory failure as a reaction to COVID-19^[4,19]^. In our study, no significant correlation was seen between blood type and clinical outcomes or death.

Most of the cases in this series have taken place before the SARS-CoV-2 infection had peaked in Brazil, and at the beginning of the pandemic, the unfamiliarity with this new disease and testing delays rendered challenging the management of the infected patients. A challenge was to recognize and properly treat these patients, making a distressing learning curve for us, as some of the symptoms and signs of COVID-19 usually overlap common postoperative findings.

Few reports until now have addressed the impact of COVID-19 in the perioperative period of cardiovascular surgery and so remains a paucity of information related to surgical procedures and patient outcomes. Sanders et al. investigated the cardiac surgery outcomes in nine United Kingdom centers during the early phase of the COVID-19 pandemic. Compared with those without COVID-19, patients who developed COVID-19 had increased mortality (24.5% *vs*. 3.5%, *P*<0.0001) and longer postoperative stay (11 days *vs*. six days, *P*=0.001). Patients who had a postoperative COVID-19 diagnosis remained in hospital for additional five days (12 days *vs*. 7 days, *P*=0.024) and presented a greatly higher mortality compared to those with a preoperative diagnosis (37.1% *vs*. 0.0%, *P*=0.005)^[21]^.

In India, Valooran et al. surveyed the early outcomes of patients who underwent cardiac surgery and who developed COVID during the in-hospital admission period. Documented perioperative COVID-19 was reported in 330 patients, with a cumulative 30-day mortality of 24.8%. The commonest cause of mortality was respiratory (33.33%), followed by multi-organ dysfunction syndrome (15.87%), and the majority of the procedures (37.1%) performed were of urgent nature^[22]^.

Interestingly, the perioperative mortality of COVID-19 patients as documented by both surveys (24.5% and 24.8%) parallels the overall mortality data reported herein (24%), as well as that from the COVID Surg Collaborative reporting from various surgical specialties (23.8%)^[2]^.

In Brazil, data from a high-volume aortic center in São Paulo compared outcomes of operated patients between the worst period of the pandemic in São Paulo (from April 1^st^ to July 31^st^, 2020) with those operated on during the same period in 2019. A nearly two-thirds reduction in operative volume was noticed, with most non-elective operations and higher mortality^[23]^.

From ours and other reports, it became clear we are dealing with a new scenario. Whereas we should continue providing urgent and elective lifesaving cardiovascular procedures to our patients, the threat of COVID-19 affecting outcomes is henceforth a reality. The COVID-19 is not supposed to disappear but remains among us as another endemic viral disease, also pending if immunity after the SARS-CoV-2 infection or a vaccine will lead to effective and lasting protection. For the cardiovascular surgeon, the decision of the appropriate surgical moment became even more sensitive in times of COVID-19 pandemic, where delays add significantly to the burden of the disease^[24]^.

Acutely, the main impact in cardiovascular surgery practice was the dramatic reduction of the operation volume, prompting the cancelation or postponement of elective surgeries, strictly limited to procedures deemed urgent or emergent, but to determine the proper decision and timing presented as a difficult challenge. Medical teams were compelled to ponder the urgency of the surgical procedures against several uncertainties and eventualities, like the risks to patients and health care personnel, the shortage of blood and derivatives, ICU beds, ventilators, and even the growing number of hospital staff on sick leave due to COVID-19.

A worldwide survey spanning 60 cardiac surgery centers has identified that cardiac surgery reduced by 50-75% during the pandemic, with a > 50% reduction in dedicated cardiac theatre rooms and ICU beds^[1]^.

Given the budgetary restriction imposed by the pandemics, a significant backlog on the waiting list for cardiac surgery is expected in the near-to-medium term and with the potential for increased mortality in this population^[25]^.

Routine preoperative COVID-19 screening became mandatory for all patients before any cardiovascular procedure, and a significant benefit of testing is the opportunity of deferring COVID-19 positive patients if they remain clinically stable, as no or uncertain actions are plausible to change the postoperative course and prognosis^[6]^.

For urgent conditions that cannot be delayed, like unstable coronary disease, the recommendation for less invasive procedures, *i.e*., percutaneous coronary intervention rather than coronary artery bypass grafting, may be questionable. The speculative lower short-term risk may be offset by inferior late outcomes.

Given the great role that the COVID-19 pandemic has been generating and its undeniable negative impact on the learning curve in the residency program in thoracic and cardiovascular surgery, a variety of technological alternatives have emerged to try to compensate for the lack of traditional academic training in medical residency towards a virtual hybrid model^[26]^.

We became extremely proud of our national healthcare system (the SUS) team working on the frontline. They were and are enduring difficult working conditions and sacrifices to care for the afflicted patients, at a times facing a shortage of personal protective equipment, many of them working overtime, their lifestyle turned upside down, but not backing out and being creative.

### Limitations

Our study has limitations. The limited number of patients included may restrict the generalization of the outcomes reported. Also, incomplete laboratory testing hinders a more robust investigation of coagulation and inflammatory markers. Future well-designed studies with larger samples will be required to elucidate the overall impact of COVID-19 in patients requiring cardiovascular surgery procedures.

## CONCLUSION

Our findings revealed that COVID-19 affecting the postoperative period of patients who underwent cardiovascular surgery is associated with a higher rate of morbidity and mortality. Delaying procedures in RT-PCR-positive patients may help improve risks of perioperative complications and death. As the disease is supposed to stay longer, efforts should be developed to improve prognosis once a SARS-CoV-2-infected patient undergoes an urgent/emergent cardiovascular operation.

**Table:** 

Authors' roles & responsibilities	
WJG	Substantial contributions to the participated in the design, data collection and interpretation, drafting, and revising of this manuscript; drafting the work or revising it critically for important intellectual content; agreement to be accountable for all aspects of the work in ensuring that questions related to the accuracy or integrity of any part of the work are appropriately investigated and resolved; final approval of the version to be published
IR	Substantial contributions to the design, data collection and interpretation, drafting, and revising of this manuscript; final approval of the version to be published
WSP	Substantial contributions to the design, data collection and interpretation, drafting, and revising of this manuscript; final approval of the version to be published
AHBP	Substantial contributions to the design, data collection and interpretation, drafting, and revising of this manuscript; final approval of the version to be published
PMSS	Substantial contributions to the design, data collection and interpretation, drafting, and revising of this manuscript; final approval of the version to be published
LAAC	Substantial contributions to the design, data collection and interpretation, drafting, and revising of this manuscript; final approval of the version to be published
MMPT	Substantial contributions to the design, data collection and interpretation, drafting, and revising of this manuscript; final approval of the version to be published
LPO	Substantial contributions to the design, data collection and interpretation, drafting, and revising of this manuscript; final approval of the version to be published
CB	Substantial contributions to the design, data collection and interpretation, drafting, and revising of this manuscript; final approval of the version to be published
IB	Substantial contributions to the design, data collection and interpretation, drafting, and revising of this manuscript; final approval of the version to be published
RSLM	Substantial contributions to the design, data collection and interpretation, drafting, and revising of this manuscript; final approval of the version to be published
NAHJ	Substantial contributions to the design, data collection and interpretation, drafting, and revising of this manuscript; final approval of the version to be published
GFV	Substantial contributions to the design, data collection and interpretation, drafting, and revising of this manuscript; final approval of the version to be published
JNRB	Substantial contributions to the design, data collection and interpretation, drafting, and revising of this manuscript; final approval of the version to be published
CAT	Substantial contributions to the design, data collection and interpretation, drafting, and revising of this manuscript; final approval of the version to be published EASM Substantial contributions to the design, data collection and interpretation, drafting, and revising of this manuscript; final approval of the version to be published
CS	Substantial contributions to the design, data collection and interpretation, drafting, and revising of this manuscript; final approval of the version to be published
AR	Substantial contributions to the design, data collection and interpretation, drafting, and revising of this manuscript; final approval of the version to be published
LMN	Substantial contributions to the design, data collection and interpretation, drafting, and revising of this manuscript; final approval of the version to be published
ARR	Substantial contributions to the design, data collection and interpretation, drafting, and revising of this manuscript; final approval of the version to be published
FKM	Substantial contributions to the design, data collection and interpretation, drafting, and revising of this manuscript; final approval of the version to be published
IEC	Substantial contributions to the design, data collection and interpretation, drafting, and revising of this manuscript; final approval of the version to be published
RCS	Substantial contributions to the design, data collection and interpretation, drafting, and revising of this manuscript; final approval of the version to be published
DLRVP	Substantial contributions to the design, data collection and interpretation, drafting, and revising of this manuscript; final approval of the version to be published
ACMJ	Substantial contributions to the design, data collection and interpretation, drafting, and revising of this manuscript; final approval of the version to be published
DSV	Substantial contributions to the design, data collection and interpretation, drafting, and revising of this manuscript; final approval of the version to be published
JHSAC	Substantial contributions to the design, data collection and interpretation, drafting, and revising of this manuscript; final approval of the version to be published
JCJ	Substantial contributions to the design, data collection and interpretation, drafting, and revising of this manuscript; final approval of the version to be published
HMRC	Substantial contributions to the design, data collection and interpretation, drafting, and revising of this manuscript; final approval of the version to be published
GK	Substantial contributions to the design, data collection and interpretation, drafting, and revising of this manuscript; final approval of the version to be published
ZSAMA	Substantial contributions to the design, data collection and interpretation, drafting, and revising of this manuscript; final approval of the version to be published
GRF	Substantial contributions to the design, data collection and interpretation, drafting, and revising of this manuscript; final approval of the version to be published
PRLL	Substantial contributions to the design, data collection and interpretation, drafting, and revising of this manuscript; final approval of the version to be published
ACF	Substantial contributions to the design, data collection and interpretation, drafting, and revising of this manuscript; final approval of the version to be published
DCB	Substantial contributions to the design, data collection and interpretation, drafting, and revising of this manuscript; final approval of the version to be published
FRHDLC	Substantial contributions to the design, data collection and interpretation, drafting, and revising of this manuscript; final approval of the version to be published
UAC	Substantial contributions to the design, data collection and interpretation, drafting, and revising of this manuscript; final approval of the version to be published
BCB	Substantial contributions to the design, data collection and interpretation, drafting, and revising of this manuscript; final approval of the version to be published
CHDM	Substantial contributions to the design, data collection and interpretation, drafting, and revising of this manuscript; final approval of the version to be published
LG	Substantial contributions to the design, data collection and interpretation, drafting, and revising of this manuscript; final approval of the version to be published
KBSP	Substantial contributions to the design, data collection and interpretation, drafting, and revising of this manuscript; final approval of the version to be published
FGJ	Substantial contributions to the design, data collection and interpretation, drafting, and revising of this manuscript; final approval of the version to be published
FRAO	Substantial contributions to the design, data collection and interpretation, drafting, and revising of this manuscript; final approval of the version to be published
RBS	Substantial contributions to the design, data collection and interpretation, drafting, and revising of this manuscript; final approval of the version to be published
ACZ	Substantial contributions to the design, data collection and interpretation, drafting, and revising of this manuscript; final approval of the version to be published
RGSM	Substantial contributions to the design, data collection and interpretation, drafting, and revising of this manuscript; final approval of the version to be published
LCBJ	Substantial contributions to the design, data collection and interpretation, drafting, and revising of this manuscript; final approval of the version to be published
RT	Substantial contributions to the design, data collection and interpretation, drafting, and revising of this manuscript; final approval of the version to be published
ABJ	Substantial contributions to the design, data collection and interpretation, drafting, and revising of this manuscript; final approval of the version to be published
LVF	Substantial contributions to the design, data collection and interpretation, drafting, and revising of this manuscript; final approval of the version to be published
PF	Substantial contributions to the design, data collection and interpretation, drafting, and revising of this manuscript; final approval of the version to be published
FO	Substantial contributions to the design, data collection and interpretation, drafting, and revising of this manuscript; final approval of the version to be published
RMJ	Substantial contributions to the design, data collection and interpretation, drafting, and revising of this manuscript; final approval of the version to be published
TCVNA	Substantial contributions to the design, data collection and interpretation, drafting, and revising of this manuscript; final approval of the version to be published
OPB	Substantial contributions to the design, data collection and interpretation, drafting, and revising of this manuscript; final approval of the version to be published
ACPS	Substantial contributions to the design, data collection and interpretation, drafting, and revising of this manuscript; final approval of the version to be published
RTBT	Substantial contributions to the design, data collection and interpretation, drafting, and revising of this manuscript; final approval of the version to be published
ILLC	Substantial contributions to the design, data collection and interpretation, drafting, and revising of this manuscript; final approval of the version to be published
ENG	Substantial contributions to the design, data collection and interpretation, drafting, and revising of this manuscript; final approval of the version to be published
PHR	Substantial contributions to the design, data collection and interpretation, drafting, and revising of this manuscript; final approval of the version to be published
LPG	Substantial contributions to the design, data collection and interpretation, drafting, and revising of this manuscript; final approval of the version to be published
NHGS	Substantial contributions to the design, data collection and interpretation, drafting, and revising of this manuscript; final approval of the version to be published
RL	Substantial contributions to the design, data collection and interpretation, drafting, and revising of this manuscript; final approval of the version to be published
SG	Substantial contributions to the participated in the design, data collection and interpretation, drafting, and revising of this manuscript; drafting the work or revising it critically for important intellectual content; agreement to be accountable for all aspects of the work in ensuring that questions related to the accuracy or integrity of any part of the work are appropriately investigated and resolved; final approval of the version to be published
